# Supplementary material for: Evolution Landscape of PiggyBac (PB) Transposon in Beetles (Coleoptera)
Source: Genes (Basel). 2025 Dec 18;16(12):1521. doi: 10.3390/genes16121521 (PMC12732532; doi:10.3390/genes16121521)
Supplement: Supplementary file 1 [file genes-16-01521-s001.zip › Supplement Materials.pdf]

SUPPLEMENTARY MATERIAL
Evolution landscape of piggyBac (PB) transposon in Beetles (Coleoptera)

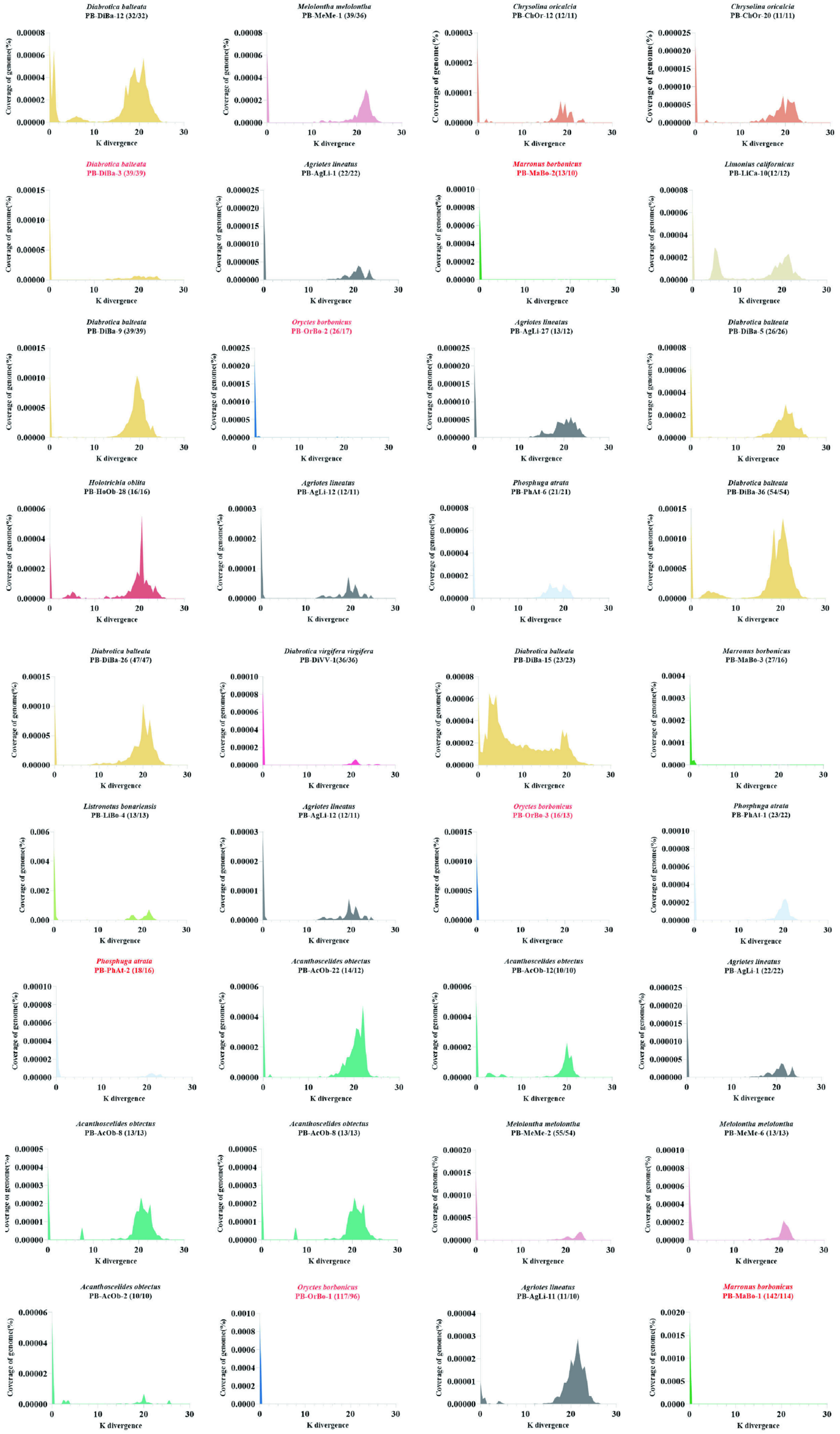

Figure S1: Evolutionary dynamics of 36 piggyBac (PB) transposons in Coleoptera. The transposon insertion ages were estimated based on K divergences as described in methods. Temporal dynamics are visualized as K divergence (X-axis) versus genomic coverage (% of total genome; Y-axis). Bracketed values indicate intact copies/total PB copies per genome. Different species are represented by distinct colors. Transposons more likely to be active are highlighted in red. (Statistical plots are sorted by sequence identity from high to low. As a supplement to the main text (Figure 4), all transposons shown have a copy number greater than 10, 36 PB transposons were selected based on sequence identity of PB copies in genome, between 80% and 99%. (For details regarding the sequences associated with the figure, please refer to Supplementary Table S3).

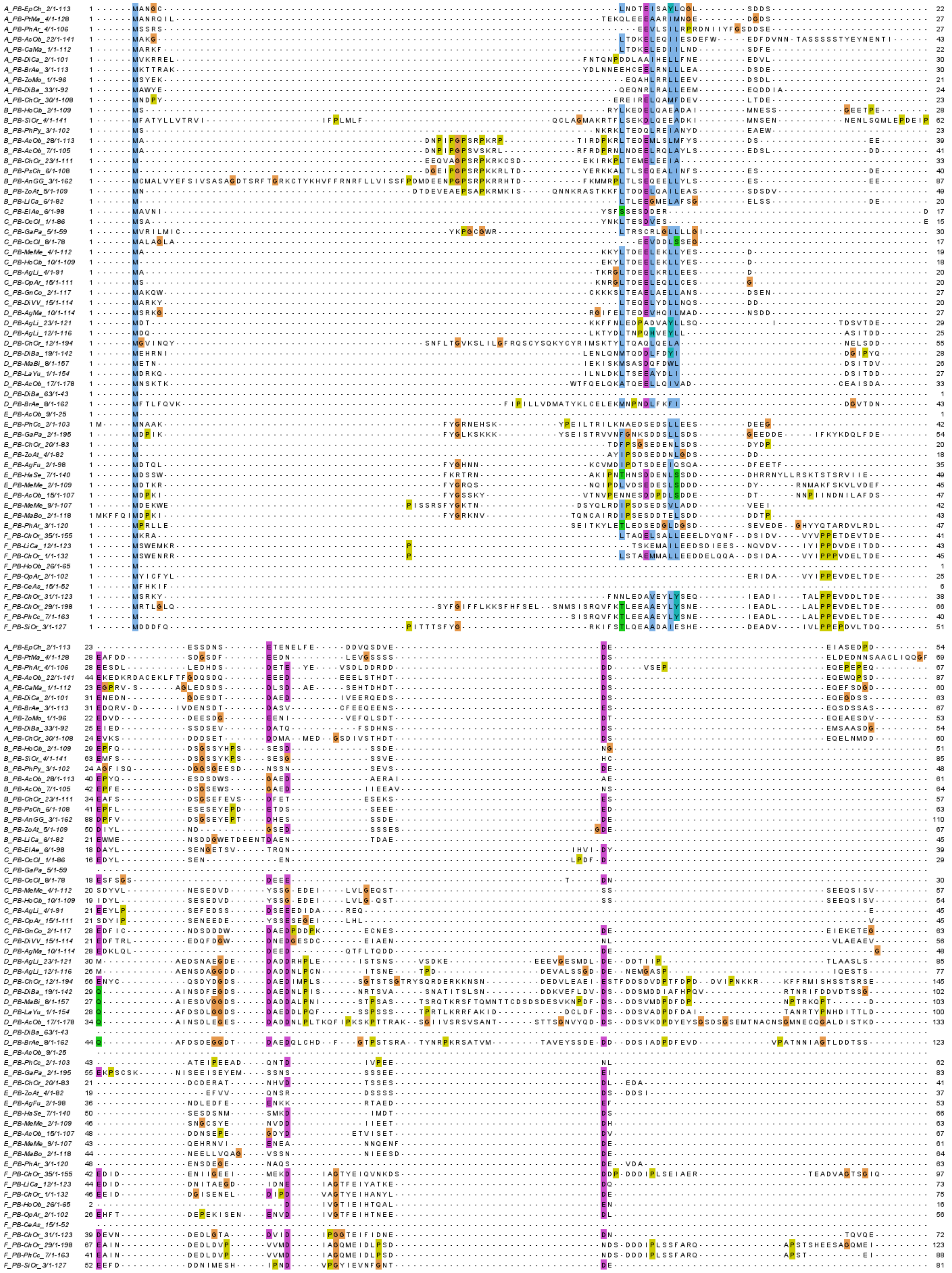

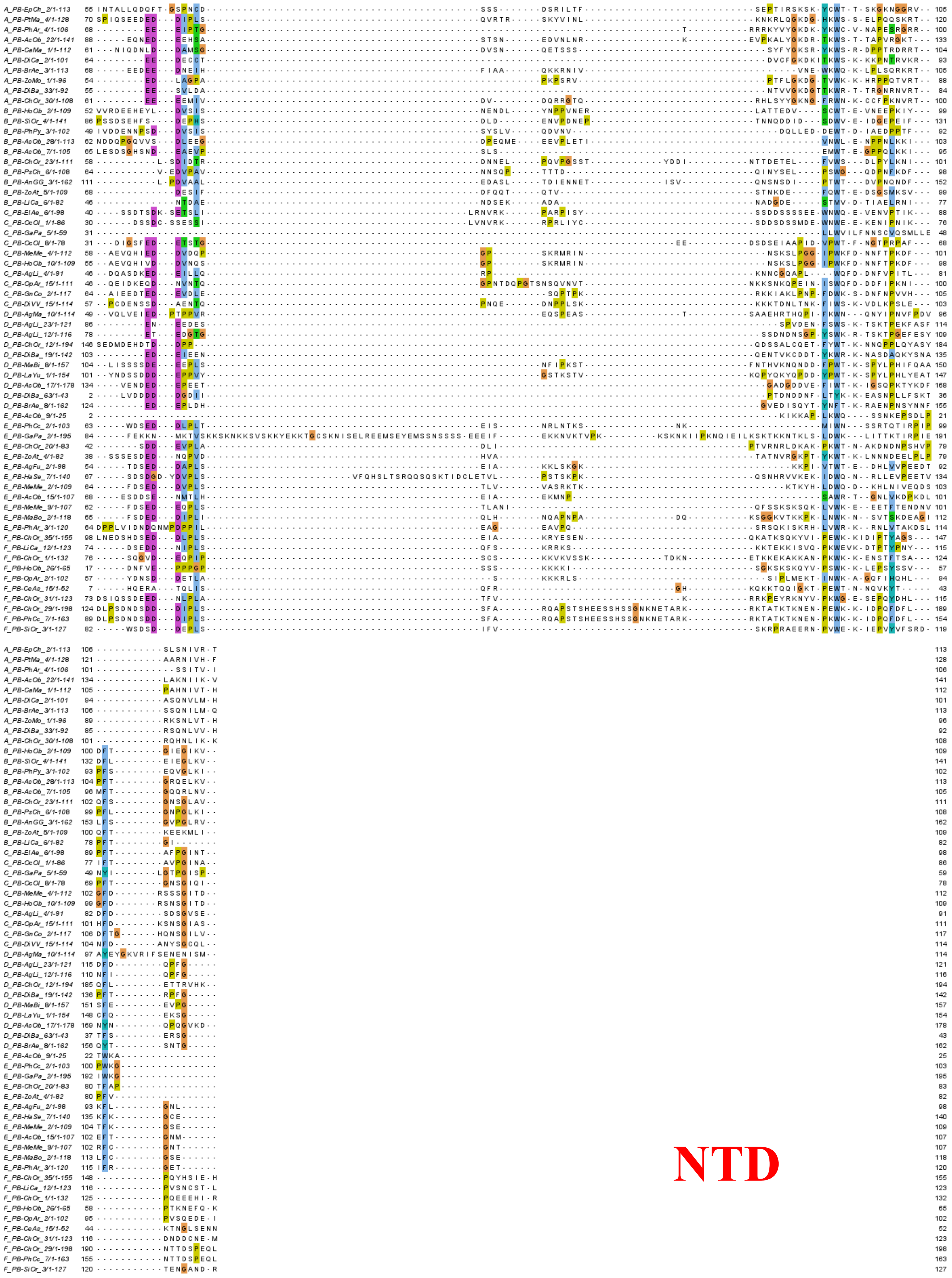
NTD

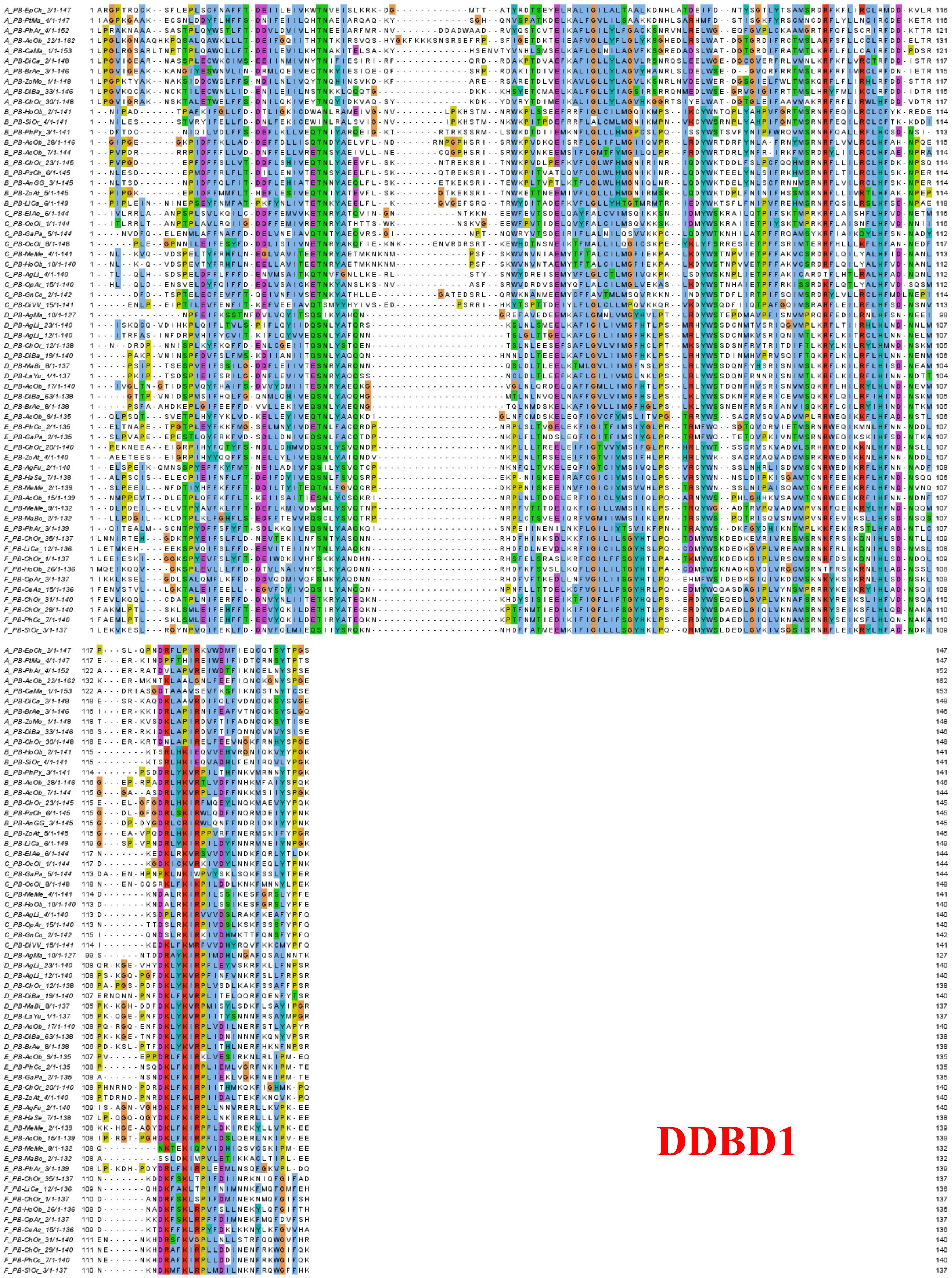

DDBD1

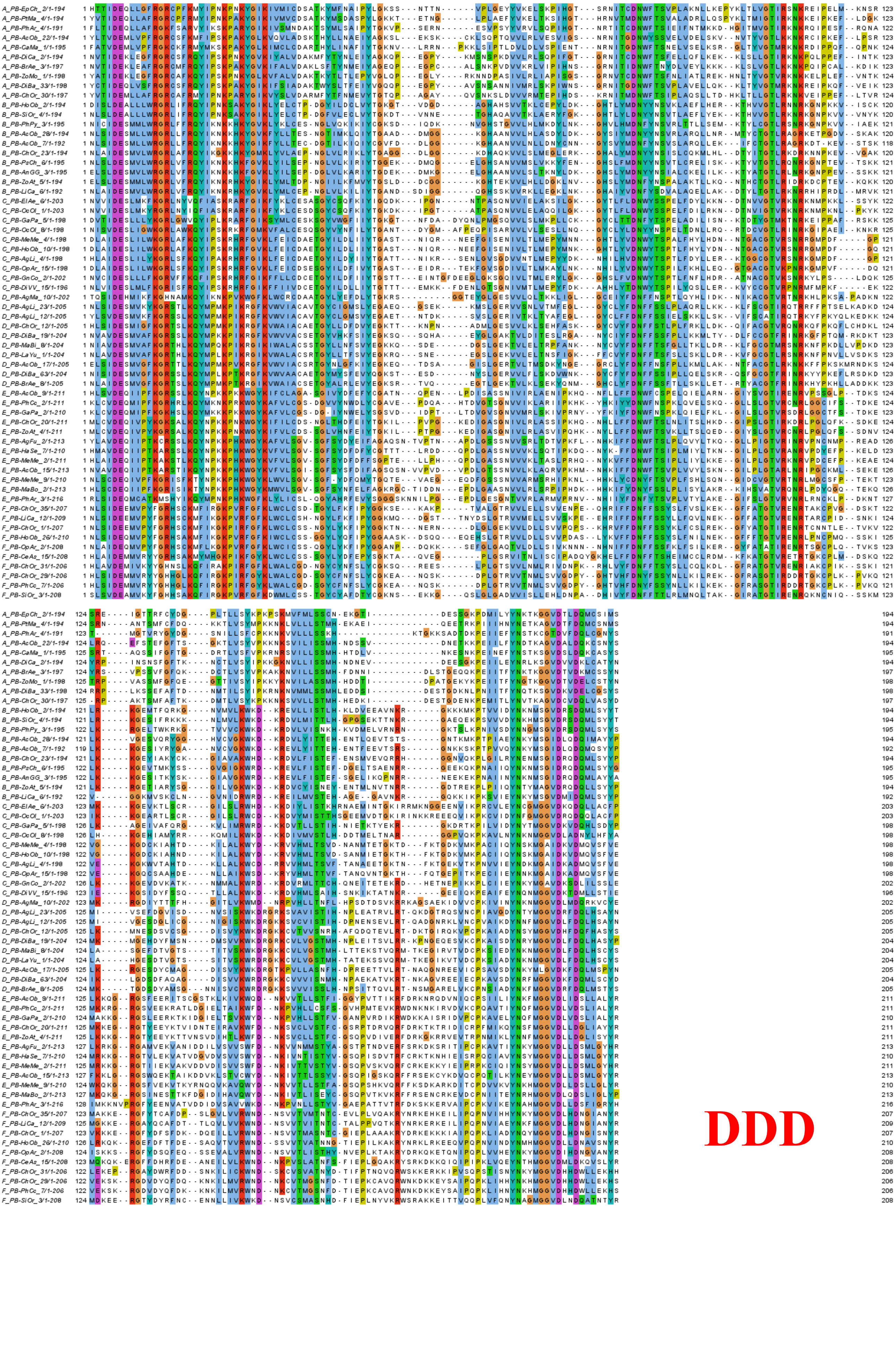
DDD

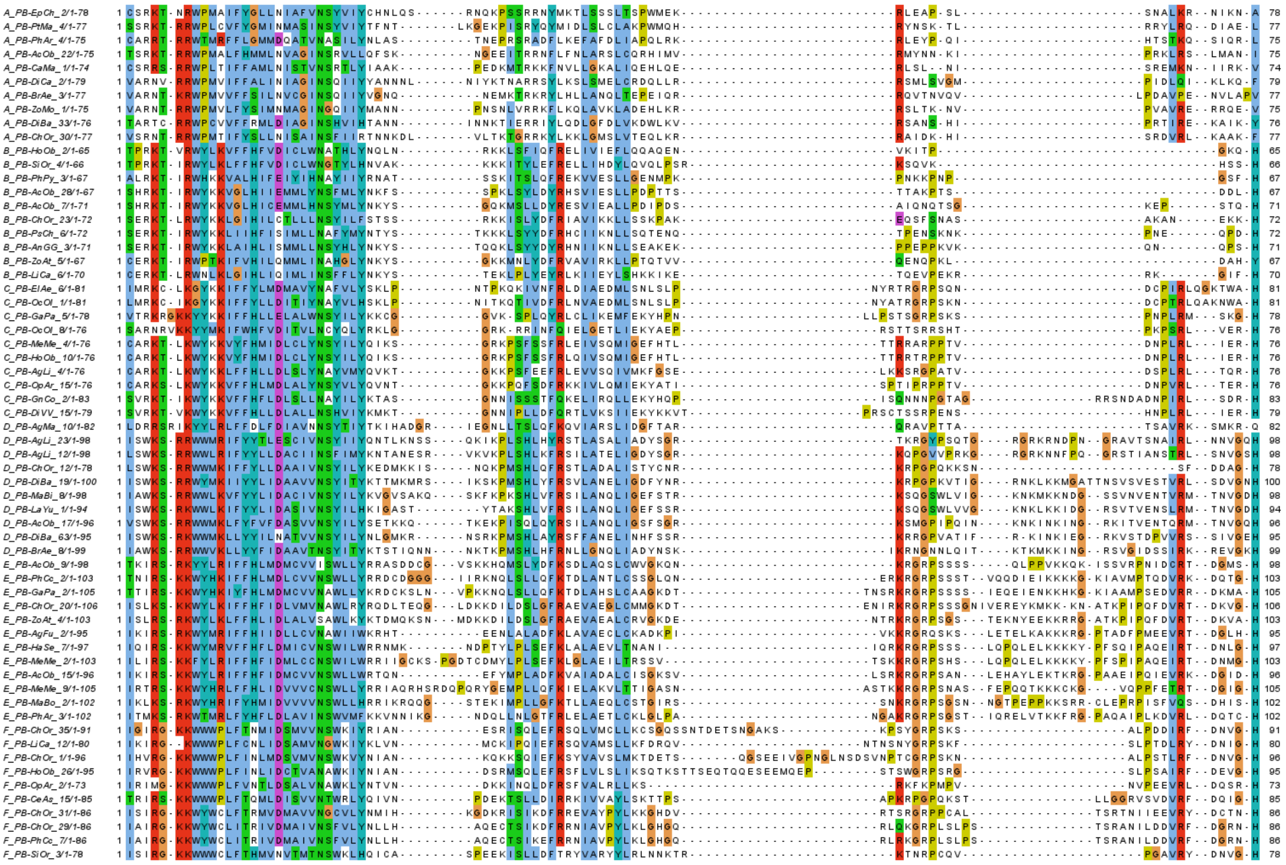

DDBD2

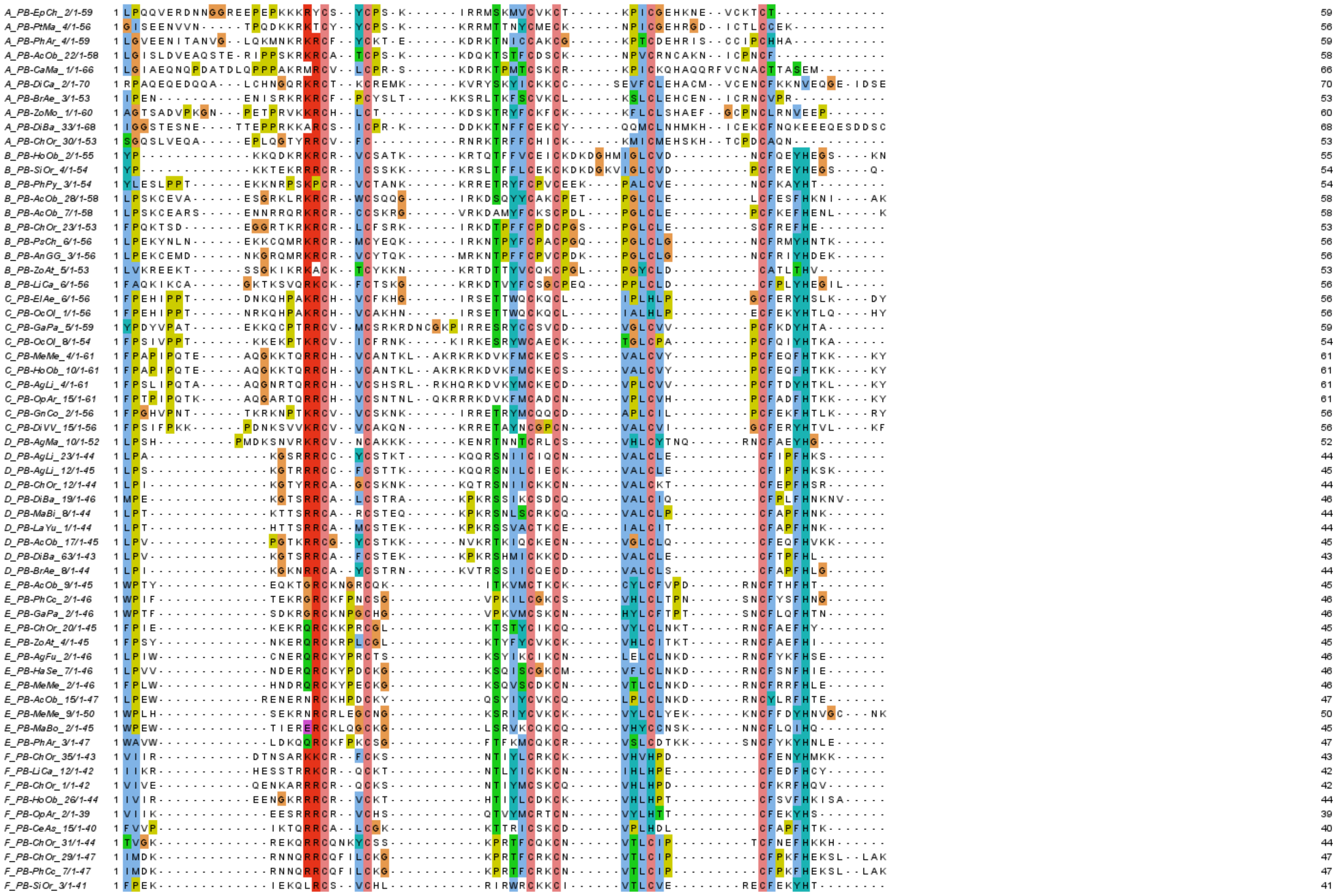

CRD

Figure S2: Alignment of amino acid sequences of partial PiggyBac transposases from different clades in Coleoptera. N-terminal domain (NTD), Dimerization and DNA binding domain 1 (DDBD1), Catalytic domain (DDD), Dimerization and DNA binding domain 2 (DDBD2), and C-terminal cysteine-rich domain (CRD). Representative sequences from each clade are shown (randomly selected). See Supplementary Table S1 for the complete set of sequences.
